# A Treatise on "Unripe" Cataract

**Published:** 1899-12

**Authors:** 


**A Treatise on "Unripe" Cataract.** By William A. M'Keown, M.D. Pp. 202. London: H. K. Lewis. 1898.

Dr. M'Keown draws attention to the length of time which is allowed to elapse before cataracts are pronounced to be ripe enough for operation. The difficulty of evacuating the lens cortex, after the nucleus has been removed, which always exists in immature and sometimes even in ripe cataracts, has especially engaged the attention of the author for many years, and has led him to devise methods for doing away with the necessity for this weary waiting. He denounces removal of the lens in its capsule, and any artificial maturation of it by massage, and regards extraction with iridectomy, or his method of injection and irrigation, as alone admissible. The various measures previous to and during operation are described in full. They do not appear to differ materially from the ordinarily accepted methods. He states, however, that cocaine is decomposed by boiling, and therefore he uses the solid hydrochlorate. He advocates in certain instances one of two special procedures. (1) Injection of a 1 per cent. saline solution inside the lens capsule by means of a fine syringe. The idea is to loosen the cortex, but it involves risk of rupturing the lens capsule and seems to have no very distinct advantage to recommend it. (2) Irrigation by introducing the point of a specially made nozzle into the lens capsule after the nucleus has been evacuted. The nozzle is connected by an indiarubber tube with a flask of saline solution held at a requisite elevation by a specially trained nurse. Statistics of 155 cases are given, and it must be said that the results are certainly good, and prove that in his hands the methods are harmless. It is, however, impossible to gather from his description the extent of immaturity of the lenses in question. Every detail of the procedure recommended is abundantly illustrated by the most beautifully executed drawings.

